# Growth performance, blood lipids, and fat digestibility of broilers fed diets supplemented with bile acid and xylanase

**DOI:** 10.5194/aab-66-451-2023

**Published:** 2023-12-21

**Authors:** Mohammed H. Alzawqari, Mustafa Shukry Atta, Abdallah Metwally, Shaimaa Selim, Mohammad A. M. Wadaan, In Ho Kim, Sungbo Cho, Hossam M. Eltahan, Mahmoud Alagawany, Rashed A. Alhotan, Ali R. Al Sulaiman, Elsayed Osman Hussein, Ahmed A. Saleh

**Affiliations:** 1 Department of Poultry Production, Faculty of Agriculture, Kafrelsheikh University, Kafr El-Sheikh 333516, Egypt; 2 Department of Animal Production, Faculty of Agriculture and Food Sciences, Ibb University, Ibb 70270, Yemen; 3 Department of Physiology, Faculty of Veterinary Medicine, Kafrelsheikh University, Kafrelsheikh 33516, Egypt; 4 Department of Nutrition and Clinical Nutrition, Faculty of Veterinary Medicine, Zagazig University, Zagazig 44511, Egypt; 5 Department of Nutrition and Clinical Nutrition, Faculty of Veterinary Medicine, University of Menoufia, Shibin El-Kom 32514, Egypt; 6 Department of Zoology, College of Science, King Saud University, P.O. Box 2455, Riyadh 11451, Saudi Arabia; 7 Animal Resource and Science Department, Dankook University, Cheonan 31116, Republic of Korea; 8 Animal Production Research Institute, Agriculture Research Center, Ministry of Agriculture, Dokki 12611, Egypt; 9 Poultry Department, Faculty of Agriculture, Zagazig University, Zagazig 44519, Egypt; 10 Department of Animal Production, College of Food & Agriculture Sciences, King Saud University, P.O. Box 2460, Riyadh 11451, Saudi Arabia; 11 Environmental Protection Technologies Institute, Sustainability and Environment Sector, King Abdulaziz City for Science and Technology, P.O. Box 6086, Riyadh 11442, Saudi Arabia; 12 Al-Khumasia For Feed and Animal Products Riyadh – Olaya – Al Aqareyah 2 – Office 705 P.O. Box 8344, Riyadh 11982, Saudi Arabia

## Abstract

This study aimed to show the effect of bile acid (BA) and xylanase (Xyl) supplementation on the growth, fat digestibility, serum lipid metabolites, and ileal digesta viscosity of broilers. A total of 720 1 d old male broilers were allocated to one of nine treatments with four replicates in each under a factorial design arrangement of three levels of BA (0 %, 0.25 %, and 0.50 %) and three levels of Xyl (0 %, 0.05 %, and 0.10 %) supplementation. The duration of the experiment was 35 d (7–42 d). Growth performance, blood lipids, fat digestibility, and ileal digesta viscosity were determined. The experimental treatments did not affect feed intake (FI) and weight gain (WG). Supplementation of BA or Xyl did not significantly ameliorate the feed conversion rate (FCR) ( p<0.05 ). The addition of BA linearly increased fat digestibility. At 7–21 d of age, the addition of BA or Xyl had a significant ( p<0.05 ) increase in serum cholesterol (Chol) but no significant difference for other serum lipid parameters in broiler chickens fed with Xyl in the starter and grower periods. However, the supplementation of 0.5 % BA at 7–21 d of age significantly increased the Chol and low-density-lipoprotein (LDL) levels. The results of this trial revealed that the supplementation of xylanases had a great effect on the degradation of arabinoxylan from wheat, which led to a relatively greater reduction in ileal digesta viscosity; it was also found that supplementation of BA significantly increased the concentration of serum lipid metabolites, whereas BA and Xyl supplementation linearly increased the fat digestibility of the birds fed wheat and tallow diets.

## 1 Introduction

Non-starch polysaccharides (NSPs) and their by-products in cereals adversely affect broilers' apparent fat digestibility (Gutiérrez-Alamo et al., 2008; Svihus, 2011; Mohamed et al., 2020; Cletus Otu et al., 2023).

Previously, wheat has had a negative reputation because of older cultivars' susceptibility to ergot and bitter constituents (Heinio et al., 2012) and its high content of fiber in the form of arabinoxylans (Bach Knudsen and Lærke, 2010), which limit unrestricted use (Jürgens et al., 2012). In particular, soluble fibers, which also increase intestinal viscosity, may reduce the digestibility of nutrients in the small intestine, a phenomenon markedly seen in broilers (Annison, 1993). Use of feed enzymes is a way to reduce intestinal viscosity, liberate entrapped nutrients, increase digestibility, and ameliorate growth retardation and digestive problems (Brufau et al., 2006). Cell-wall-degrading enzymes for wheat-based diets are successfully used in poultry. Furthermore, the use of xylanases, which traditionally are developed for wheat, is effective. The decrease in lipid digestibility is not merely due to the reduction of digestive enzymes' secretion (Nir et al., 1993) and bile acids but also the binding of non-starch soluble polysaccharides to bile acids (Smits et al., 1998; Dibner and Richards, 2004; Abudabos et al., 2019). These components cannot be digested by birds as they cannot produce different enzymes (Dibner and Richards, 2004; Saleh et al., 2017, 2018, 2023). Bird diets have abundant exogenous enzymes that can hydrolyze NSPs (Cho and Kim, 2013). Consequently, the use of NSP-degrading enzymes is a popular method for improving the feed efficiency of wheat-based feed in an attempt to minimize the effect on the producer margins (Bedford, 2000; Choct, 2006; Inayah et al., 2022). There is ample evidence that adding enzymes to broilers that contain wheat, barley, oats, and triticale by hydrolysis of soluble xylan and beta-glucan decreases the viscosity of the digestive content and improves the quality of nutrient digestibility (Pettersson et al., 1990). Elevated viscosity in the intestine changes several mechanisms in the bird (Alzawqari et al., 2016). Additionally, increasing intestinal viscosity reduces the incorporation of pancreatic enzymes with dietary nutrients. The hydrolysis of nutrients by different digestive enzymes is inhibited by transfer to the epithelial surface, which in turn reduces the digestion of the nutrients (Fengler and Marquardat, 1988). Scott (2002) recorded an average increase in metabolizable energy by 9 % by including enzymes in wheat-based diets. Other researchers have also confirmed this rise in metabolizable energy (Marshall and John, 2013; Saleh et al., 2023). Although viscosity's effect on broilers' performance factors is evident, the relationship between variation in grain and viscosity quality is not fully understood. Most researchers agree that the majority of beneficial effects (50 %–70 %) of dietary enzymes are due to decreased viscosity in the digestive tract, as enzymes enhance the diet's digestibility (Sun et al., 2015; Saleh et al., 2018).

Bile acids are essential for emulsification and help to absorb fats (Lai et al., 2018). Young birds have a fixed ability to make biliary salts (Bansal et al., 2021). Decreasing the bile acid cycle induces a remarkable lowering in the size of bile acids and increases the bile acid concentration. This reduction has a negative effect on the fat emulsion, which reduces fat absorption. Long-chain digestion of unsaturated fatty acids is strongly impacted relative to unsaturated fatty acids (Abedi et al., 2014; Eltahan et al., 2023). Increasing the ileal digesta viscosity and increasing microbial activity may decrease the conjugation of bile acids with one of the glycine or taurine amino acids, which has a negative effect on the fat emulsion (Engberg et al., 2000). The supplementation of bile salts had a beneficial effect on the apparent digestibility of fat (Maisonnier et al., 2003; Kirrella et al., 2021). It is hypothesized that dietary bile acid and xylanase addition is expected to have favorable impacts on broiler chickens. Thus, the purpose of the current study was to determine the effects of bile acid (BA) and xylanase (Xyl) supplements on the performance, fat digestibility, serum lipid metabolites, and ileal digestive viscosity of broiler chickens fed wheat and tallow diets.

## 2 Material and methods

### 2.1 Birds, design, and management

The study was approved by the Ethics Committee of Local Experimental Animals Care Committee and conducted following the guidelines of Kafrelsheikh University, Egypt (no. 4/2016 EC). All precautions were followed to minimize suffering during the entire experimental period.

The xylanase (Xyl) enzyme was obtained from Endofeed WDC and GNC Bioferm Inc., Bradwell, SK, Canada. This product's minimum activity of beta-glucanase and arabinoxylanase was 440 and 1200 units per gram, respectively. The BA product which was used in this trial was prepared from fresh bile acid, which was collected from the gall bladder of oxen at a local slaughterhouse. Thereafter, homogenates of bile acids were filtered through a coarse nylon mesh, concentrated, and dried under a high vacuum at low temperature and immediately stored at - 20 ${}^{\circ }$ C to prevent decomposition (Alzawqari et al., 2016). Colors, as well as other physical and chemical characteristics, are shown in Table 1. BA was checked to be free from contamination such as *E. coli* and *Salmonella* spp.

**Table 1:** Physical and chemical characteristics of dried bile acid (BA) collected from broilers.

Item	Characteristic
Color powder	brownish to yellow
Color in solution	yellow-beige
Solubility (%)	100
pH	5.5–7.5
Ash (%)	15
Water (%)	5

A total of 720 1 d old male broilers (Ross 308) bought from a local hatchery were held in floor pens ( 1×1 m) containing wood shavings along the trail. Birds were divided into nine groups with four replicates in each under a factorial design arrangement of three concentrations of bile acid (BA) (0 %, 0.25 %, and 0.50 %) by three levels of xylanase (Xyl) (0 %, 0.05 %, and 0.10 %) supplementations. The chicks were given the pre-starter diet for 1 week before the experiment, and the experimental diets were administered starting from the second week. Chickens were given the experimental diets for starter (7–21 d) and grower (22–42 d) periods. The diets were isocaloric, with similar nutrients, and were wheat-based in formulation, with 5 % and 15 % tallow for starter and grower diets, respectively, that met the recommended levels for broiler chicks by the NRC (1994) and were provided in mash form (Table 2).

**Table 2:** Composition and calculated nutrient contents of the experimental diets. ME: metabolizable energy.

Ingredient (%)	Starter	Grower
	(7–21 d)	(22–42 d)
Wheat	43.13	49.30
Yellow corn	10.00	–
Soybean meal (44 %, CP)	24.62	22.31
Gluten meal (62 %, CP)	5.82	1.75
Wheat bran	7.67	8.46
Dicalcium phosphate	1.44	0.95
Limestone	1.23	1.34
Vit. and min. premix ${}^{1}$	0.50	0.50
Salt	0.38	0.25
Tallow	5.00	15.00
L-Lysine	0.08	0.07
DL-Methionine	0.13	0.07
Total	100.00	100.00
Calculated analysis ${}^{2}$		
ME (kcal kg ${}^{-1}$ )	2950	3050
Crude protein (%)	21.20	18.44
Crude fat (%)	7.04	7.10
Crude fiber (%)	4.21	4.27
Calcium (%)	0.92	0.83
Available phosphorus (%)	0.42	0.32
Sodium (%)	0.18	0.14
Arginine (%)	1.17	1.06
Lysine (%)	1.14	0.92
Methionine + cysteine (%)	0.83	0.66
Tryptophan (%)	0.28	0.26
Linoleic acid	1	1

${}^{1}$ Supplied per kilogram of diet: vitamin A, 10 000 IU; vitamin D ${}_{3}$ , 9790 IU; B ${}_{12}$ , 20 µ g; riboflavin, 4.4 mg; calcium pantothenate, 40 mg; niacin, 22 mg; choline, 840 mg; biotin, 30 µ g; thiamine, 4 mg; zinc sulfate, 60 mg; manganese oxide, 60 mg. ${}^{2}$ Calculated according to the NRC (1994).

**Table 3:** The effect of supplemental bile acid (BA) and xylanase (Xyl) levels on the performance of male broiler chickens during 7–42 d of age.

Main effects			FI (gram/bird/day)			BWG (gram/bird/day)			FCR (g feed : g gain)		
			7–21 d	22–42 d	7–42 d	7–21 d	22–42 d	7–42 d	7–21 d	22–42 d	7–42 d
BA (%)											
0.00			52.7 ${}^{a}$	150.9	111.2	33.6 ${}^{b}$	69.4	52.2	1.71 ${}^{a}$	2.17	2.14 ${}^{a}$
0.25			55.5 ${}^{b}$	149.4	109.5	35.1 ${}^{a}$	71.3	53.9	1.58 ${}^{b}$	2.10	2.03 ${}^{b}$
0.50			57.0 ${}^{\mathrm{ab}}$	151.1	111.0	34.3 ${}^{\mathrm{ab}}$	72.2	54.2	1.67 ${}^{a}$	2.09	2.06 ${}^{b}$
Xyl (%)											
0.00			56.9 ${}^{\mathrm{ab}}$	151.8	111.4	34.0	70.0	52.7	1.68	2.16 ${}^{a}$	2.13 ${}^{a}$
0.05			55.7 ${}^{b}$	148.9	110.3	34.0	70.1	52.8	1.63	2.13 ${}^{\mathrm{ab}}$	2.07 ${}^{\mathrm{ab}}$
0.10			57.4 ${}^{a}$	150.7	111.0	35.0	72.8	54.7	1.65	2.07 ${}^{b}$	2.03 ${}^{b}$
SEM ±		0.79	2.21	1.39	0.72	1.86	1.11	0.03	0.04	0.04	
Interaction effects											
BA	×	Xyl									
0.00		0.00	57.5	149.6	110.4	33.7	67.6	51.0	1.70	2.20	2.18
0.25		0.00	54.9	151.0	110.2	34.6	71.0	53.5	1.60	2.13	2.10
0.50		0.00	58.2	154.8	113.8	33.9	71.6	53.7	1.73	2.15	2.13
0.00		0.05	57.0	152.7	112.0	33.3	71.7	53.5	1.70	2.13	2.10
0.25		0.05	54.1	145.9	106.8	34.6	69.9	53.0	1.55	2.10	2.00
0.50		0.05	56.1	148.2	109.0	34.0	68.6	51.9	1.65	2.18	2.10
0.00		0.10	58.2	150.5	111.2	34.0	69.0	52.2	1.73	2.18	2.15
0.25		0.10	57.6	151.3	111.4	36.2	72.9	55.2	1.60	2.08	2.00
0.50		0.10	56.5	150.4	110.4	34.9	76.5	56.9	1.63	1.95	1.95
Source of variance			P value								
BA (%)			0.015	0.583	0.269	0.055	0.195	0.089	0.0004	0.099	0.002
Xyl (%)			0.041	0.288	0.153	0.161	0.133	0.051	0.328	0.050	0.006
BA × Xyl			0.103	0.173	0.109	0.921	0.100	0.102	0.309	0.057	0.076

FI: feed intake. BWG: body weight gain. FCR: feed conversion ratio. SEM: standard error of the mean. Means in the same column with no superscript letters or a common superscript letter following them are not significantly different ( p<0.05 ).

### 2.2 Data collection

Body weights of broilers were individually recorded every week. However, feed intake was measured daily (on a pen basis) throughout the experimental period, and the feed conversion ratio (FCR) was calculated during 7–21, 22–42, and 7–42 d of age. Each cage was considered as one replicate, and a total of four replicates were used for each dietary treatment. FCR = replicate feed intake divided by replicate body weight gain (Vilela et al., 2021). Daily mortality was recorded, and the performance was corrected.

The fat digestibility was measured using the method of Fenton and Fenton (1979) and Scott et al. (1998). On days 21 and 42, one bird was obtained that was selected from each replicate; the birds were weighed, and winged vein blood samples were taken (3 mL). An autoanalyzer (Autolab, BT 3500, biotechnica instruments, Rome, Italy) was used to assess serum cholesterol (Chol), triglyceride (TG), high-density lipoprotein (HDL), and low-density lipoprotein (LDL) (Tankson et al., 2002). At ages 21 and 42, one bird per replicate was selected, labeled, and weighed; the ileum was aseptically dissected; and digesta viscosity was measured, as stated by Lázaro et al. (2004).

### 2.3 Statistics

The resulting data were tested for normality distribution using Shapiro–Wilk and Kolmogorov–Smirnov tests, and then analysis of variance (ANOVA) was conducted by applying the SAS software generalized linear model (GLM) procedure (SAS Institute, 2008) as a 3×3 factorial treatment arrangement, including BA and Xyl dietary supplementation as the primary consequences and interactions. The means of treatment were analyzed at a statistical level by Tukey's p<0.05 test.

## 3 Results

### 3.1 Performance

Table 3 shows the effect of supplemental bile acid (BA) and xylanase (Xyl) on performance from 7–42 d of age. From the results, it can be inferred that there were no significant effects on performance between the treatments during the experiment ( p<0.05 ). However, adding 0.5 % BA at 7–42 d of age significantly enhanced the FCR. Inclusion of 0.1 % of Xyl also significantly improved the FCR over 22–42 and 7–42 d of age compared to the control ( p<0.05 ) treatment. In contrast, no interaction was observed over the entire experiment between BA and Xyl on the performance of birds.

### 3.2 Fat digestibility

The effect of BA and Xyl supplementation on fat digestibility in diets of male broiler chickens based on wheat and tallow is illustrated in Table 4. Fat digestibility increased significantly with increased BA levels ( p<0.05 ). Including Xyl at the age of 7–21 d did not affect the digestibility of the diets. Although fat digestibility increased significantly ( p<0.05 ) compared to other treatments by adding 0.05 % Xyl at 22 to 42 d of age, the difference between 0.05 % and 0.1 % Xyl levels for fat digestion ability was not substantial. In addition, the interaction effects between 0.5 % of BA and 0.05 % of Xyl at 22 to 42 d of age were higher fat digestibility ( p<0.05 ).

**Table 4:** The effect of supplemental bile acid (BA) and xylanase (Xyl) levels on fat digestibility of male broiler chickens during 7–42 d of age.

Main effects			Fat digestibility (%)	
			7–21 d	22–42 d
Interaction effects				
BA	×	Xyl		
0.00		0.00	44.97	49.67 ${}^{d}$
0.25		0.00	55.07	64.13 ${}^{\mathrm{cd}}$
0.50		0.00	76.57	76.37 ${}^{\mathrm{abc}}$
0.00		0.05	53.20	59.37 ${}^{\mathrm{cd}}$
0.25		0.05	71.37	75.53 ${}^{\mathrm{abc}}$
0.50		0.05	76.77	82.73 ${}^{a}$
0.00		0.10	36.50	64.80 ${}^{\mathrm{bcd}}$
0.25		0.10	69.13	59.63 ${}^{\mathrm{cd}}$
0.50		0.10	84.07	81.97 ${}^{\mathrm{ab}}$
Source of variance			P value	
BA (%)			0.0001	0.0001
Xyl (%)			0.190	0.017
BA × Xyl			0.129	0.044

Means in the same column with no superscript letters or a common superscript letter following them are not significantly different ( p<0.05 ).

### 3.3 Blood constituents

Table 5 demonstrates the effect of the BA and Xyl supplement on the serum lipid metabolites of broiler chickens. At 7–21 d of age, the addition of BA or Xyl had a significant ( p<0.05 ) increase in serum cholesterol (Chol) but no significant difference for other serum lipid parameters in broiler chickens fed with Xyl in the starter and grower periods. However, the supplementation of 0.5 % BA at 7–21 d of age significantly increased the Chol and LDL levels. Adding 0.5 % BA at 22 to 42 d of age increased the Chol, HDL, and LDL levels compared to the control treatment. The interaction between BA and Xyl was not found in serum lipid metabolites in broilers.

**Table 5:** The effect of supplemental bile acid (BA) and xylanase (Xyl) levels on serum lipid metabolites (SB) of male broiler chickens during 7–42 d of age.

Main effects			SB (mg dl ${}^{-1}$ )							
			7–21 d				22–42 d			
			Chol	TG	HDL	LDL	Chol	TG	HDL	LDL
BA (%)										
0.00			134.00 ${}^{b}$	130.33	89.11	40.89 ${}^{b}$	114.11 ${}^{a}$	51.33	78.78 ${}^{a}$	32.78 ${}^{b}$
0.25			143.22 ${}^{b}$	131.55	78.56	49.33 ${}^{\mathrm{ab}}$	93.78 ${}^{b}$	48.56	63.78 ${}^{b}$	19.56 ${}^{b}$
0.50			163.33 ${}^{a}$	133.11	78.33	56.56 ${}^{a}$	120.00 ${}^{a}$	48.47	79.33 ${}^{a}$	32.56 ${}^{a}$
Xyl %										
0.00			132.33 ${}^{b}$	131.33	79.00	42.67	107.11	49.44	72.11	42.22
0.25			150.44 ${}^{a}$	131.33	84.56	49.67 ${}^{a}$	104.78	47.56	71.67	25.67
0.50			157.56 ${}^{a}$	132.22	82.44	54.44 ${}^{a}$	116.00	51.65	78.11	26.89
SEM ±			7.06	2.44	5.94	6.78	6.66	6.38	5.55	3.12
Interaction effects										
BA	×	Xyl								
0.00		0.00	123.67	136.00	71.67	33.33	123.00	54.33	83.00	27.66
0.25		0.00	125.33	130.00	74.67	42.00	87.00	57.33	58.67	18.33
0.50		0.00	154.66	128.00	90.67	52.67	111.33	35.67	74.67	17.33
0.00		0.05	138.33	132.33	80.00	39.00	94.33	40.00	66.67	19.67
0.25		0.05	149.33	130.33	85.67	48.67	92.67	52.67	65.33	23.00
0.50		0.05	163.67	130.67	88.00	61.33	127.33	50.00	83.00	34.33
0.00		0.10	146.00	130.67	84.00	50.33	125.00	59.67	86.67	26.00
0.25		0.10	155.00	134.33	74.67	57.33	101.67	35.67	67.33	17.33
0.50		0.10	171.67	131.66	88.67	55.67	1211.33	59.33	80.33	37.33
Source of variance			P value							
BA (%)			0.0005	0.371	0.063	0.036	0.0003	0.905	0.004	0.0002
Xyl (%)			0.0027	0.842	0.524	0.130	0.122	0.851	0.307	0.586
BA × Xyl			0.8526	0.298	0.526	0.720	0.028	0.090	0.165	0.066

Chol: cholesterol. TG: triglyceride. HDL: high-density lipoprotein. LDL: low-density lipoprotein. Means in the same column with no superscript letters or a common superscript letter following them are not significantly different ( p<0.05 ).

### 3.4 Viscosity of the ileal digesta

Table 6 shows the effect of adding BA and Xyl to the viscosity of the ileal digesta. The inclusion of BA did not affect broiler chickens' ileal digesta viscosity between 7 and 42 d of age ( P>0.05 ). The addition of Xyl, however, significantly reduced the ileal digesta viscosity ( p<0.05 ). No effects on the viscosity of the ileal digesta from contact between BA and Xyl were observed.

**Table 6:** The effect of supplemental bile acid (BA) and xylanase (Xyl) levels on the ileal digesta viscosity of male broiler chickens during 7–42 d of age.

Main effects			Viscosity (cPs ${}^{1})$	
			7–21 d	22–42 d
BA (%)				
0.00			1.36	1.38
0.25			0.43	1.54
0.50			1.35	1.35
Xyl (%)				
0.00			1.61 ${}^{a}$	1.70 ${}^{a}$
0.05			1.31 ${}^{b}$	1.32 ${}^{b}$
0.10			1.22 ${}^{b}$	1.24 ${}^{b}$
SEM ±			0.13	0.15
Interaction effects				
BA	×	Xyl		
0.00		0.00	1.64	1.75
0.25		0.00	1.68	1.72
0.50		0.00	1.51	1.64
0.00		0.05	1.18	1.21
0.25		0.05	1.35	1.49
0.50		0.05	1.40	1.26
0.00		0.10	1.26	1.18
0.25		0.10	1.26	1.42
0.50		0.10	1.14	1.11
Source of variance			P value	
BA (%)			0.795	0.368
Xyl (%)			0.012	0.010
BA × Xyl			0.790	0.911

cPs: centipoises. Means in the same column with no superscript letters or a common superscript letter following them are not significantly different ( p<0.05 ).

## 4 Discussion

Energy ingredients account for around 65 %–70 % of the overall energy costs in the broiler industry (Saleh et al., 2019). Additionally, fat is not the only source of energy and fatty acids in poultry feed; other cereal grains can also provide energy. The fat digestibility in the young chick is restricted, as it has poor levels of bile production (Tancharoenrat et al., 2013; Eltahan et al., 2023). Synthetic bile acid was estimated to improve fat digestion in young broilers' diets according to this aim. Different studies have shown an improvement in broilers' dietary bile acid feed efficiency (Siyal et al., 2017). Alzawqari et al. (2011) found that the dietary supplementation of tallow with ox bile at 5 g kg ${}^{-1}$ resulted in a higher weight gain and an improved feed conversation ratio. Similarly, supplementation of bile salts significantly increased broilers' body weight gain (Maisonnier at el., 2003). Adding 0.5 % purified chenodeoxycholic acid did not affect feed intake (Diego-Cabero et al., 2015; Piekarski et al., 2016; Bansal et al., 2021). In addition, Huang et al. (2014) tested taurine with animal fats in birds aged 1 week but not in adult birds, where the impact could have occurred. Our results, on the other hand, were consistent with similar findings by Wang et al. (2005). In both studies, enzyme supplementation to broilers rye or wheat-based diets was reported to have significantly improved the feed intake and feed conversion ratio.

Conversely, Huang et al. (2014) reported no pronounced additional enzyme effect. Furthermore, Cowieson and Masey (2013) reported that xylanase supplementation was the second most successful strategy for improving the feed conversion ratio in broilers. Almirall et al. (1995) found that exogenous enzyme supplementation in broiler feed enhanced the FCR, and this effect was related to improving digestibility and minimizing intestinal viscosity. Moreover, improving growth performance by adding NSP enzymes could be explained by their involvement in reducing digesta viscosity and modifying gut microbiota by improving beneficial microbes (Saleh et al., 2018; Abdel-Latif et al., 2017; Saleh et al., 2023). On the other hand, some of the positive effects on efficiency may have been attributed to improved fat digestibility (Silva and Smithard, 2002; Inayah et al., 2022). Likewise, in this study, dietary supplemental bile acids improved feed conversion into broilers but did not affect average feed intake.

Bile acids play a crucial role in enhancing the digestion efficiency of fat (Hofmann, 2009). Adding fresh bovine bile to the broiler diet showed a linear increase of 53 to 83 percent in the digestibility of tallow fat (Lai et al., 2018). In our previous studies, we found that adding dried ox bile into broiler chicken diets significantly increased the apparent digestibility of the fat (Alzawqari et al., 2010, 2011, 2016; Saleh et al. 2019). Furthermore, Ohtani and Yayota (2002) found that by adding bile salts to broiler chicken diets at 14 d of age and measuring ileal digestibility using the ileum method or by stool collection, the apparent digestibility of adipose significantly increased ( p<0.001 ).

Moreover, Maisonnier et al. (2003) found that the inclusion of bile salts was reported to have a positive effect on the apparent fat digestibility of broiler chicken diets. Saleh et al. (2019) recently reported that the inclusion of dietary supplements with Xyl and Abf (arabinofuranosidase) enzymes significantly increased crude fat digestibility ( p<0.05 ). This enhancement may be related to improving nutrient digestibility by supplementing the Xyl and Abf enzymes. One of the many factors for a pronounced decrease in fat digestibility is the presence of wheat pentosan due to the viscous properties of wheat pentosan in the gastrointestinal tract, which can reduce the rate of digestive enzyme exposure (Ward, 1983) and simply the rate of nutrient release through the digestive tract. Secondly, bile salts in young chicks seem to be a limiting factor (Gomez and Pollen, 1976; Meng and Slominski, 2005; Cowieson and Masey, 2008; Cozannet et al., 2017). Therefore, a decrease in bile salt secretion reduces fat absorption in young chicks, and this effect is more pronounced in young chickens when the dietary fat is tallow. Third, the interactions between bile salts and the microbial population in the gastrointestinal tract decrease fat digestibility.

The absorption of saturated fatty acids depends more on the bile salts being secreted than unsaturated fats (Kussaibati, 1982). In addition, NSP increases the diet of the gut microbial population, which reduces the emulsification power of bile salts due to their conversion to bile acids (Campbell et al., 1983). Ox bile supplementation substantially increased Chol and LDL components of serum lipid (Alzawqari et al., 2010, 2011, 2016; Inayah et al., 2022). Other researchers documented that supplementing cholic acid in young chicks' diets containing 0.8 % cholesterol induced a small increase in the cholesterol concentration of chickens (Hegsted et al., 1960; Abudabos et al., 2019). Additionally, Crespo and Esteve-Garcia (2003) reported that total serum cholesterol concentrations in broilers fed with tallow diets were higher than those containing sunflower or cottonseed oil. Also, they showed that decreased bile acid secretion could have a limited effect on the digestibility and absorption of fat, particularly high-chain fatty acids. The findings by Ketels (1994) showed that the highest digestibility in the treatment of tallow fat contained 0.50 % dried bovine bile relative to other treatments ( p<0.05 ). These results indicate that more bile acids did not affect the ability to transport cholesterol from tissues into the liver. Therefore, bile acids play an important role in lipid digestibility by elevating the function of enzymes and promoting the digestion of dietary fats (Siyal et al., 2017). However, this disagreed with Ge et al. (2018), who confirmed that dietary bile acid supplementation decreased serum TG and LDL. Hermier (1997) stated that there was no substantial difference from the control group in the impact of enzyme supplementation on serum cholesterol and HDL. In addition, adding the enzyme to broiler chickens' diet did not significantly affect cholesterol and HDL (Zarghi and Golian, 2009).

Current results agree with our previous findings that the application of dissected ox bile to broiler feed did not influence ileal digestive viscosity at 21 and 42 d (Alzawqari et al., 2010, 2011, 2016). Additionally, Maisonnier et al. (2003) suggested that adding bile salts to diets did not influence the viscosity of the intestines. Carbohydrase enzymes, which have an enzymatic system for decomposing branch chains and subunits of arabinoxylanase and beta-glucanases, can reduce the intestinal leachate viscosity if used in diets containing a high concentration of wheat (Bedford, 2000). Moreover, several studies concluded that adding an enzyme to broiler chicken diets containing a high concentration of NSPs and tallow showed more positive effects on the enzyme and minimized the intestinal content viscosity (Almirall et al., 1995; Saleh et al., 2019; Abudabos et al., 2019).

## 5 Conclusions

The results of the present study indicate that dietary BA and Xyl wheat-based supplementation of broiler chicken tallow diets has a significant ( p<0.05 ) impact on growth performance, including FCR and serum lipid metabolites. Adding Xyl with or without BA enhanced the digestibility of fat substantially ( p<0.05 ). The addition of Xyl, in turn, had a significant effect ( p<0.05 ) on reducing broiler chickens' ileal digesta viscosity. However, more work needs to be done to better understand the other effects of supplementation of BA and Xyl in tallow and wheat diets and under different conditions.

## Data Availability

The data presented in this study are available free of charge for any user upon reasonable request from the corresponding author.
